# Proteins Structure Models in the Evaluation of Novel Variant (C.472_477del) in the *MOCS2* Gene

**DOI:** 10.3390/diagnostics10100821

**Published:** 2020-10-14

**Authors:** Aleksandra Jezela-Stanek, Witold Blaz, Artur Gora, Malgorzata Bochenska, Katarzyna Kusmierska, Jolanta Sykut-Cegielska

**Affiliations:** 1 Department of Genetics and Clinical Immunology, National Institute of Tuberculosis and Lung Diseases, 01-138 Warsaw, Poland; 2 Clinical Department of Neonatology and NICU, Saint Jadwiga the Queen Clinical Provincial Hospital No2, 35-301 Rzeszow, Poland; witekblaz@yahoo.com; 3 Tunneling Group, Biotechnology Centre, Silesian University of Technology, 44-100 Gliwice, Poland; a.gora@tunnelinggroup.pl; 4 Clinical Department of Pediatric Neurology, Saint Jadwiga the Queen Clinical Provincial Hospital No2, 35-301 Rzeszow, Poland; malgorzatabochenska@interia.pl; 5 Department of Inborn Errors of Metabolism and Paediatrics, Institute of Mother and Child, 01-211 Warsaw, Poland; Katarzyna.kusmierska@imid.med.pl (K.K.); Jolanta.cegielska@imid.med.pl (J.S.-C.)

**Keywords:** molybdenum cofactor deficiency type B, *MOCS2* gene, crystal protein structure

## Abstract

(1) Background: Molybdenum cofactor deficiency type B (MOCODB, #252160) is a rare autosomal recessive metabolic disorder characterized by intractable seizures of neonatal-onset, muscular spasticity, accompanying with hypouricemia, elevated urinary sulfite levels and craniofacial dysmorphism. Thirty-five patients were reported to date. (2) Methods: Our paper aimed to delineate the disease genotype by presenting another patient, in whom a novel, in-frame variant within the *MOCS2* gene was identified. (3) Results: Exome sequencing led to the identification of a novel variant in the *MOCS2* gene-c.472_477del of unknown significance (VUS). (4) Conclusions: To prove the clinical significance of the mentioned variant, analysis of the possible mutation consequences on molecular level with the use of the available crystal structure of the human molybdopterin synthase complex was of great importance. Moreover, a potential pathomechanism resulting from a molecular defect was presented, giving original insight into the current knowledge on this rare disease, including treatment options.

## 1. Introduction

Molybdenum cofactor deficiency type B (MOCODB, #252160) is an autosomal recessive metabolic disorder characterized by intractable seizures of neonatal-onset, muscular spasticity, accompanied by hypouricemia, elevated urinary sulfite levels and craniofacial dysmorphism. It first came to medical attention in 1980 [1]. Affected children show severe neurologic complications, which may lead to early death, and rarely (only seven cases described to date) present with a milder form with global developmental delay without seizures [2,3]. The severe manifestation of the disease is characterized by neonatal-onset, intractable seizures, severe feeding difficulties, accompany by progressive neurological deterioration (opisthotonos, hypertonicity, spastic quadriplegia) and cystic leukomalacia in brain MR. The milder phenotype is defined with longer surveillance and first occurrence of seizures after the neonatal period. In both types, dysmorphic facial features may be noted, as a long face with frontal bossing, hypertelorism, puffy cheeks and long philtrum. The disorder results from decreased activity of sulfite oxidase (SUOX; EC 1.8.3.1) and xanthine dehydrogenase (XDH; EC 1.17.1.4 and 1.17.3.2), which are molybdenum cofactor-dependent for their activity, while molybdenum cofactor (MoCo) is essential also for aldehyde oxidase activity (AOX1; EC 1.2.3.1). It is mainly sulfite oxidase deficiency that will lead to the severe clinical presentation.

Molybdenum cofactor (MOCS2) is encoded by the *MOCS2* gene, localized on chromosome 5q11.2. Its biosynthesis is, however, a multistep process, involving the synthesis of precursor Z by proteins encoded by *MOCS1* (603707), conversion of precursor Z to molybdopterin (MPT) by MPT synthase (MOCS2), and attachment of molybdenum to the dithiolene moiety of MPT by gephyrin (GPHN; EC 2.10.1.1 and 2.7.7.75) [4,5]. MOCS2 is bicistronic, overlapping open reading frames (ORFs) which encode MOCS2A and MOCS2B, the two subunits of MPT synthase [6], which implicates its functioning and molecular diagnostics of MOCS2 deficiency.

In this paper, we seek present another patient, in whom a novel, in-frame variant within the *MOCS2* gene was identified. We aim to delineate the MOCS2 phenotype and give evidence of the clinical significance of the novel variant.

## 2. Clinical Description and Methods 

The patient was born from second pregnancy by Caesarean section (due to polyhydramnios, fetal heart rate anomalies in cardiotocography) in the 38th week with a birth weight of 3960 g, and head circumference of 37 cm (>97th percentile). In the Apgar scale, he received 7/7/10/10 points. Irregular breathing, pale grey skin, and increased muscle tone were noted; the saturation was 65% and there was mild metabolic acidosis in umbilical cord blood. Mechanical ventilation was used to stabilize the child’s condition. During pregnancy, the mother was diagnosed with hypothyroidism, treated with levothyroxine; fetal hiccups were noted. The family history for miscarriages, deaths in early childhood, and neurological and metabolic diseases were negative. The child’s parents are unrelated; the proband has a healthy, 2-year-old sister.

Physical examination revealed facial dysmorphism (such as coarse features with puffy cheeks, bitemporal narrowing, deep-set eyes, long philtrum, dysplastic auricles), long and overlapping fingers, large feet, pectus excavatum, swelling of the eyelids and lumbosacral region. At 30 min after the birth, deterioration in the general condition and grunting drops in desaturation were observed with subsequent diagnosis of congenital pneumonia. Difficulties in feeding (lack of sucking), muscular tension disorders, epileptic seizures, and breathing difficulties with an abnormal crying further complicated the perinatal period. Convulsions with drops in saturation occurred on the first day of life. Despite the phenobarbital implementation, polymorphic epileptic seizures, limb myoclonus, breathing disorders, sometimes with desaturation, were still observed. Within the following days, the child became inactive and flaccid. Abnormal spontaneous movements of the extremities were observed, such as nonrhythmic waving, pedaling, and increased muscle tone, especially in upper extremities. Reduced muscular tension was accompanied by areflexia. Since seizures were still uncontrolled, levetiracetam was additionally introduced to the treatment with initially good effect. During the hospitalization, muscle tone gradually increased, and elbow and knee joints contractures appeared.

In the second month of life, the child gradually became more reactive, but still presented clearly an impoverished spontaneous motor activity. At times, the boy opened his eyes, but with no eye contact and did not follow with his eyes. He was fed with a nasogastric tube. Physically, the skin was pale, pasty, head circumference was still above the 97th percentile, and prominent cranial sutures were noted. The axial hypotonia and lower limb spasticity were observed; tendon hyperreflexia appeared. In the neurological examination, the traction test was negative, no sole reflex on both sides, no Babinski sign bilaterally, weak grasping reflexes, no Moro reflex. Periodical restlessness, crying, and hyperekplexia were observed, as well as epileptic seizures, mainly myoclonic. Thus, in addition to phenobarbital and levetiracetam, valproic acid was later introduced. Due to tachycardia, propranolol was included in the treatment. Hypothyroidism requiring substitution with levothyroxine was also diagnosed. Because of his severe and worsening clinical condition, the child was transferred to a home hospice.

The laboratory and imagine assessment were summarized in Table 1.

In order to verify the clinical diagnosis—deficiency of molybdenum cofactor—taken in terms of disease manifestation and laboratory result, genetic testing was warranted. However, given the nonspecific features (macrocephaly at birth, hypertrophic cardiomyopathy and lack of cystic leukomalacia in brain imagining), we decided on exome sequencing.

### 2.1. Methods

#### 2.1.1. Molecular Analysis

Whole exome sequencing (WES) was performed in 3billion, Inc (Seoul, South Korea), using genomic DNA isolated from the patient’s whole blood with informed consent obtained from the patient’s parents; in the light of the applicable law, there was no requirement for ethics committee review. The captured genomic regions (exons of approx. 22,000 genes) were sequenced using a NovaSeq platform (Illumina, San Diego, CA, USA) and aligned to the reference sequence (NCBI genome assembly GRCh37; accessed in February 2009), as previously described [7]. The mean depth of coverage was 100-fold, with 99.2% coverage higher than 10-fold. Sanger sequencing of the identified variant was performed for the patient.

#### 2.1.2. Crystal Structures Analysis

The crystal structure of human molybdopterin synthase complex (PDB ID: 5MPO [8]) and molybdopterin synthase from *Staphylococcus aureus* complexed with precursor Z (PDB ID: 2QIE [9]) were downloaded from the Protein Data Bank [10]. Protein structures were aligned and analyzed using PyMol software (PyMol Version 2.0, Schrödinger LLC, New York, NY, USA). The potential effects of substitutions at 158 and 159 positions in the molybdopterin synthase catalytic subunit were examined by the PredictSNP webserver (https://loschmidt.chemi.muni.cz/predictsnp/ access date: 11 May 2020) [11].

## 3. Results

### 3.1. Genetic Result

WES led to the identification of a variant in the *MOCS2* gene—c.472_477del (Table 2, Figure S1). The results of the predicted effect of mutations at position 158 and 159 of the catalytic subunit of molybdopterin synthase complex obtained by the PredictSNP server [11] are shown (Table S1).

### 3.2. Structural Analysis

**Figure 2:**
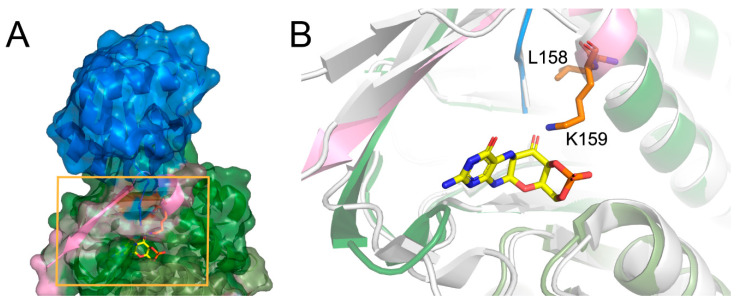
The crystal structure of human molybdopterin synthase (**A**). The molybdopterin synthase sulphur carrier subunit (blue) and molybdopterin synthase catalytic subunit (green and pink) are shown in cartoon and surface representation. The close-up on carrier subunit bounding cavity (**B**). The Leu158, Lys159 amino acids are shown as orange sticks, the C-terminus of molybdopterin synthase catalytic subunit is shown in pink. The white cartoon shows the aligned structure of the molybdopterin synthase from *S. aureus* complexed with precursor Z (yellow).

There is no crystal structure of the protein carrying the described deletion; however, the analysis of the native conformation of the molybdopterin synthase and comparison with the structure from *Staphylococcus aureus* complexed with precursor Z shed light on the consequences of the deletion. Human molybdopterin synthase complex was crystallized as a dimer constituted of two heterodimers. Each heterodimer consists of molybdopterin synthase sulfur carrier subunit and molybdopterin synthase catalytic subunit. The Leu158 and Lys159 deletion occurs in the catalytic subunit. The Leu158 and Lys159 residues are located at the end of the last helix, close to the C-terminus of the subunit, and together with the C-terminus, they participate in the molybdopterin synthase sulfur carrier subunit binding (Figure 2A). Moreover, the comparison of the structures of molybdopterin synthases from human and *Staphylococcus aureus* suggests that the Lys159 residue is essential for precursor Z binding (Figure 2B).

## 4. Discussion

The molybdenum cofactor deficiency is an ultra-rare autosomal recessive disease, characterized by rapidly progressive and severe neurological damage, sometimes misdiagnosed as hypoxic-ischemic encephalopathy. The clinical and molecular characteristics of molybdenum cofactor deficiency due to *the MOCS2* variant have been recently extensively reviewed by Arican et al. [2]. The presented group encompassed 35 patients with proven molecular etiology and 29 different pathogenic variants identified in the *MOCS2* gene.

Our proband’s history and clinical features, in terms of neonatal seizures and the biochemical test results, are consistent with the previous descriptions. That prompted us to test for a deficiency of molybdenum cofactor, while neonatal-onset, refractory epilepsy, feeding difficulties, and facial dysmorphism were the most suggestive phenotypic features. The proband presented, however, some unique characteristics, not consistent with molybdenum cofactor deficiency, like macrocephaly at birth, hypertrophic cardiomyopathy (restrictive) with elevated troponin and lack of cystic leukomalacia in brain imagining. Under such circumstances, the performance of genetic diagnostics, to a greater extent than targeted testing (of the *MOCS2* gene only), was justified. As a result, the initial clinical diagnosis, supported by undetectable serum uric acid, very low serum homocysteine, and positive sulfite dipstick results (the test definitely showed a high concentration of sulfites in the fresh urine; sulfocysteine was not measured, but the purine and pyrimidine profile in urine showed very high xanthine concentration), was finally verified in whole-exome sequencing analysis. It revealed a variant of unknown significance in the *MOCS2* gene. The homozygous variant c.472_477del on *MOCS2* deletes nucleotides ‘CTTTTA’ from chromosome5 52396264, which does not change frameshift in the protein-coding sequence. It has been reported with an extremely low frequency in the large population cohorts (GenomAD). This in-frame deletion in the non-repeat region can change the length of proteins and disrupt protein function. This variant is classified as of uncertain significance according to the recommendation of the American College of Medical Genetics (ACMG) and Association for Molecular Pathology (AMP) guideline. The child’s clinical features were, however, highly consistent with the disease. Additionally, taking into account the homozygous state of this variant, we suggest it as being causative. Referring to the additional clinical characteristics, not reported in previous cases, their significance as features of molybdenum cofactor deficiency type B needs to be verified based on additional cases with the same genetic etiology (genotype).

We have performed an analysis of the crystal structure of the human molybdopterin synthase complex to support this statement. The Leu158 and Lys159 are localized at the end of the last helix of the catalytic subunit, just before the 13 aa of the C-terminus. This region of the molybdopterin synthase has dual functionality. It binds the sulfur carrier subunit and the precursor Z. The alignment of the crystal structure of human molybdopterin synthase with the one of *Staphylococcus aureus* complexed with precursor Z reflects that Lys159 corresponds to Leu116 of (*S. aureus*) as an essential residue for proper binding of the precursor Z.

As a consequence of the mutation, the Leu158 will be replaced by Ala160, and Lys159 by Lys161. Luckily, the deleted Lys159 should be replaced by Lys161, and thus its role can be preserved (a similar replacement of the key amino acid was observed, for example, in D-amino acid oxidase, where Tyr314 repossesses the function of the Tyr55, eliminated by the Tyr55Ala mutation and modifies the enzyme-substrate specificity) [12]. Since the second one replaces the crucial lysine, one could expect that the precursor Z binding can still occur; however, the protein–ligand affinity can be already modified. However, in this case, the situation is more complicated. The second lysine residue (123 in *S. aureus* and 166 in human) is essential for precursor Z binding, and this crucial residue is located at the C-terminus. It is highly probable that the deletion of two residues can modify the position of the second lysine.

Moreover, it will have consequences on the folding of the C-terminus and can result in changes in binding affinity of the sulfur carrier subunit to the catalytic one. Thus, the described deletion could have significant consequences for both the heterodimer and the active complex formation, which may suggest another mechanism of pathogenicity of the c.472_477del variant in the *MOCS2* gene. As results from predictSNP have shown (Table S1), no deletion analysis is possible, but the report clearly indicates that any mutation in this area has consequences. However, to verify which functionality is mostly disturbed, the precursor binding or carrier subunit binding, further study has to be considered.

## 5. Conclusions

In this paper, we give clinical and structural evidence of pathogenicity of the novel, in-frame variant c.472_477del in the *MOCS2* gene. We emphasize the need for rapid genetic diagnosis, using next-generation sequencing—rapid WES (whole-exome sequencing)—in metabolic diseases, especially in cases with an unclear clinical picture or when the prognosis is poor. The described mutation modifies the binding cavity of the catalytic subunit, and the preliminary structural analysis shows that two potential processes that can be distorted. We can speculate that if the heterodimer formation is preserved, the deficiency caused by abnormal precursor Z binding could perhaps be limited by such chemical modification of the precursor Z molecule that would restore native complex properties. The second potential mechanism of deficiency, improper carrier subunit binding, would be much more challenging to reverse.

## Figures and Tables

**Figure 1:**
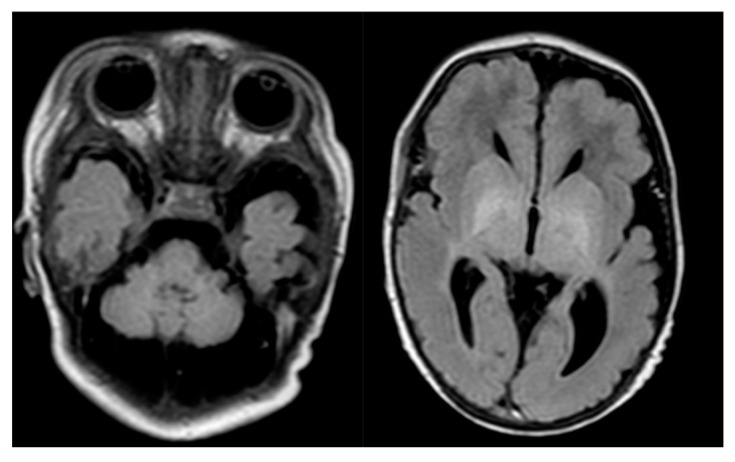
Proband’s brain MR. Cerebellar hypoplasia and dilated cerebellar fluid spaces.

**Table 1:** Abnormal results of laboratory and imagine assessment of our Proband.

Test	Result
uric acid/serum	not detectable
homocysteine/serum	<1.00 umol/L
Sulfite dipstick	positive
brain US	3rd day after birth— vague brain structure marginal widening of the occipital corners of both lateral ventricles (normal Evans index) slightly accelerated flows in the sagittal sinus 4th week of life— extensive malacic lesions within the frontal and parietal cortex symmetrically with prevalence on the right side and hyperechogenic areas around tegmentum and globus pallidus bilaterally 2nd month of life— extensively disseminated porencephaly within the hemispheres and the subcortical regions— extensive disseminated brain necrosis and low-pressure hydrocephalus
brain MR (5th day after birth, Figure 1)	cerebellar hypoplasia and dilated cerebellar fluid spaces slightly enlarged cerebral fluid spaces in the left temporal region a discrete band of enhanced signal in T1-dependent images and FLAIR within one of the grooves of the left frontal lobe (after the bleeding?) the supratentorial ventricular system symmetrically widened within the lateral ventricle (Evans index - 0.36)
EEG	neonatal period— localized changes with a tendency to generalize 2nd month of life— almost continuous right- or left-sided localized burst-suppression pattern
ECHO	hypertrophic cardiomyopathy, non-restrictive
other	elevated levels of troponin and NT-proBNP, hypothyroidism

FLAIR: fluid-attenuated inversion recovery; NT-proBNP: N-terminal pro b-type natriuretic peptide; US: ultrasound imaging; EEG: electroencephalography; ECHO: echocardiography.

**Table 2:** Genetic result of our patient. Nomenclature of the variant following the guidelines of the Human Genome Variation Society using NM_004531.4 as a reference cDNA sequence and NP_004522.1 as a protein sequence.

Gene	MOCS2
Variant nomenclature	
cDNA Level:	NM_004531.3: c.472_477del
gDNA Level:	Chr5(GRCh38):g.53100435_53100440del
Protein Level:	NP_004522.1: p.(Leu158_Lys159del)
Variant type	inframe deletion
Zygosity	homozygous
Allele frequency *	-
Classification	VUS
Disease	Molybdenum cofactor deficiency B (autosomal recessive)

* Allele frequency is based on genomes in the gnomAD database; VUS variant of unknown significance.

## Supplementary Materials

The following are available online at https://www.mdpi.com/2075-4418/10/10/821/s1, Figure S1: WES results; Table S1: SNP analysis.

## References

[1] Johnson J.L., Waud W.R., Rajagopalan K.V., Duran M., Beemer F.A., Wadman S.K. (1980). Inborn errors of molybdenum metabolism: Combined deficiencies of sulfite oxidase and xanthine dehydrogenase in a patient lacking the molybdenum cofactor. Proc. Natl. Acad. Sci. USA.

[2] Arican P., Gencpinar P., Kirbiyik O., Bozkaya Yilmaz S., Ersen A., Oztekin O., Olgac Dundar N. (2019). The Clinical and Molecular Characteristics of Molybdenum Cofactor Deficiency Due to MOCS2 Mutations. Pediatr. Neurol..

[3] Scelsa B., Gasperini S., Righini A., Iascone M., Brazzoduro V.G., Veggiotti P. (2019). Mild phenotype in Molybdenum cofactor deficiency: A new patient and review of the literature. Mol. Genet. Genom. Med..

[4] Reiss J., Dorche C., Stallmeyer B., Mendel R.R., Cohen N., Zabot M.T. (1999). Human molybdopterin synthase gene: Genomic structure and mutations in molybdenum cofactor deficiency type B. Am. J. Hum. Genet..

[5] Reiss J., Hahnewald R. (2011). Molybdenum cofactor deficiency: Mutations in GPHN, MOCS1, and MOCS2. Hum. Mut..

[6] Leimkuhler S., Freuer A., Araujo J.A., Rajagopalan K.V., Mendel R.R. (2003). Mechanistic studies of human molybdopterin synthase reaction and characterization of mutants identified in group B patients of molybdenum cofactor deficiency. J. Biol. Chem..

[7] Seo G.H., Taeho K., Park J., Lee J., Kim S., Won D.G., Oh A., Lee Y., Choi I.H., Choi J. Pilot Study of Evidence: High Diagnostic Yield and Clinical Utility of Whole Exome Sequencing Using an Automated Interpretation System for Patients with Suspected Genetic Disorders. https://www.biorxiv.org/content/10.1101/628438v3.

[8] Kopec J., Bailey H., Fitzpatrick F., Strain-Damerell C., Oberholzer A.E., Williams E., Burgess-Brown N., von Delft F., Arrowsmith C., Edwards A. Crystal Structure of Human Molybdopterin Synthase Complex. https://www.rcsb.org/structure/5MPO.

[9] Daniels J.N., Wuebbens M.M., Rajagopalan K.V., Schindelin H. (2008). Crystal structure of a molybdopterin synthase-precursor Z complex: Insight into its sulfur transfer mechanism and its role in molybdenum cofactor deficiency. Biochemistry.

[10] Rose P.W., Prlić A., Altunkaya A., Bi C., Bradley A.R., Christie C.H., Costanzo L.D., Duarte J.M., Dutta S., Feng Z. (2017). The RCSB protein data bank: Integrative view of protein, gene and 3D structural information. Nucleic Acids Res..

[11] Bendl J., Stourac J., Salanda O., Pavelka A., Wieben E.D., Zendulka J., Brezovsky J., Damborsky J. (2014). Predict SNP: Robust and accurate consensus classifier for prediction of disease-related mutations. PLoS Comput. Biol..

[12] Subramanian K., Góra A., Spruijt R., Mitusińska K., Suarez-Diez M., Martins Dos Santos V., Schaap P.J. (2018). Modulating D-amino acid oxidase (DAAO) substrate specificity through facilitated solvent access. PLoS ONE.

